# Inter Laminar Shear Strength of Flax-Glass Hybrid Polymer Composites for Automotive Frame: Numerical Modelling and Experimental Analysis

**DOI:** 10.3390/ma18163852

**Published:** 2025-08-17

**Authors:** Tegginamath Akshat, Michal Petru, Rajesh Kumar Mishra

**Affiliations:** 1 Department of Machine Parts and Mechanism, Faculty of Mechanical Engineering, Technical University of Liberec, Studentská 1402/2, 461 17 Liberec, Czech Republic; akshattm93@gmail.com; 2 Department of Material Science and Manufacturing Technology, Faculty of Engineering, Czech University of Life Sciences Prague, Kamycka 129, Suchdol, 165 00 Prague, Czech Republic

**Keywords:** hybrid composites, inter laminar shear strength (ILSS), finite element analysis (FEM), glass, flax, scanning electron microscopy (SEM)

## Abstract

This study deals with the mechanical performance in the case of hybrid polymer composites developed from sandwiched reinforcements using natural fibre and glass fibre-based fabrics. The composites developed by using different combinations and arrangements of the glass and flax fabrics were tested for the interlaminar shear strength (ILSS). Finite element analysis based on ANSYS was used to determine the ILSS for the hybrid composites. Further, experimental testing of the ILSS was carried out in order to validate the predicted performance. The comparison of simulated values with the tested values showed percentage error values ranging from 0.106% to 6.25%. The minor error between the tested and simulated values can be due to the presence of very small imperfections in the composite, like the presence of voids, which could potentially be introduced in the composite while manufacturing the samples. Microscopic analysis confirmed the fracture in between the layers and interfacial debonding between the fibre and the matrix. It was found that the flax fibre tends to break earlier as compared to the glass component, which has much better mechanical performance. The findings are important for understanding the performance of hybrid composites in real loading conditions in automotive frames and other similar applications.

## 1. Introduction

In general, composite materials have an edge due to the fact that the composite materials can satisfy the various needs required to make the final product acceptable. The high specific strength, low maintenance cost, low density [1,2,3,4], high stiffness and lightweight characteristics [5] of the fibre-reinforced composites have led to their widespread utilisation in various engineering fields [1]. Traditionally, the internal components and frames in automobiles are made from metals, but these parts now can be replaced with the fibre-reinforced composites. These composites have found application not only in the automobile industry but also in the aviation sector and the marine sector as well, where the weight of the components has been reduced drastically in addition to the superior mechanical properties [5]. In the following years the utilisation of these composites is predicted to increase exponentially [6,7], as this would fall in line with the idea of reducing the impact on the environment due to the usage of traditional materials and lead to the development of sustainable, eco-friendly composite materials which utilise natural fibres as reinforcing materials [8,9,10].

In recent years the utilisation of hybrid composites has taken centre stage, owing to the extreme versatility of hybrid composites in their characteristics, and the range of applications in which hybrid composites can be utilised is vast [5]. In addition to this, the mechanical and physical properties can be modified to a very high degree to produce a suitable hybrid composite [10]. In order to create ecofriendly and sustainable composite materials, natural fibres like flax, sisal, jute, hemp, etc., have been combined with high-performance fibres like Kevlar, carbon, glass, etc. [11].

Upon comparing the mechanical performance of the natural fibres with high-performance fibres, it is highlighted that the natural fibres are inherently weaker. In order to circumvent this glaring difference in mechanical performance, the outer layers of the composite can be made from these high-performance fibres, and the other layer can be made from the natural fibres. Thus, a hybrid composite with sustainable and ecofriendly material can be produced, which has two different reinforcing materials [12]. The challenges and drawbacks associated with a composite made from a single type of reinforcement can be overcome by utilising a hybrid composite where multiple reinforcing fibres can be utilised. The hybrid composites which are produced can then be used in the aviation sector, marine sector, and automotive sector, along with making blades for wind turbines and manufacturing various household items.

Fibres such as glass, Kevlar, basalt, carbon, etc. can be used to design frames and components which can be used to replace the existing components and frames made using traditional materials. The frames made using high-performance fibres are converted into composites by using resins. These frames are usually mechanically sturdy and ultra-lightweight in nature. To predict the behaviour of these composite frames, advanced computational tools can be used [13,14,15,16,17,18,19,20].

**Figure 1:**
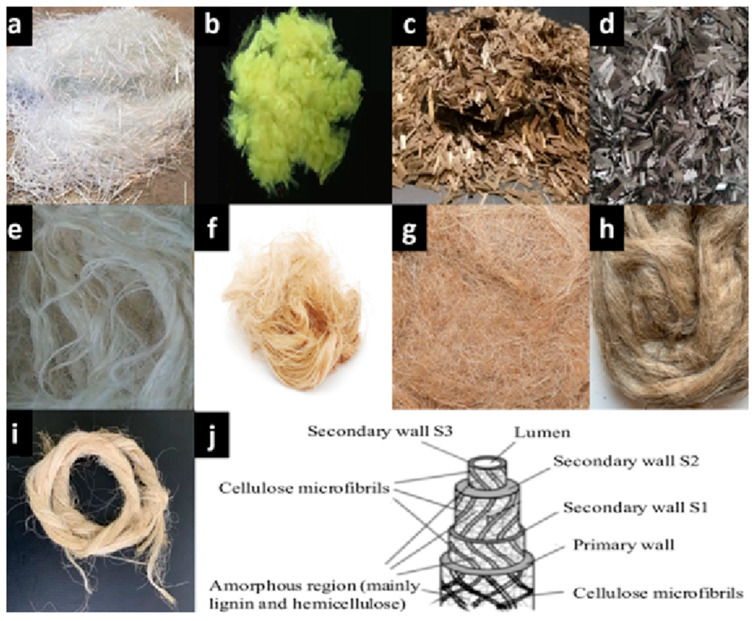
(**a**) Glass, (**b**) Kevlar, (**c**) Basalt, (**d**) Carbon, (**e**) Sisal, (**f**) Hemp, (**g**) Jute, (**h**) Flax, (**i**) Banana fibres and (**j**) Microstructure of flax [47].

The nonabrasive nature, biodegradability, lightweight, zero toxicity, lower costs and ease of machinability are the advantages that the natural fibres offer over the synthetic fibres [21,22,23,24,25,26]. Natural fibre-reinforced composites based on fibres obtained from plants are commonly manufactured using sisal, hemp, jute, flax, banana, etc. [27,28]. Various authors have investigated in detail the physical and mechanical properties of arrowroot, sisal, kenaf, jute, pinecone, bamboo, coir and hemp fibre composites [29,30,31,32,33,34]. When synthetic fibres are added along with the natural fibres while producing natural fibre-based composites, the thermal and mechanical properties of the natural composite are greatly improved, along with it giving rise to a hybrid composite. Studies have also been conducted on tensile properties [35,36,37,38], interlaminar shear [39], flexural [39], damping [40], and impact [41,42,43,44,45,46] to determine the effect of hybridisation. The various synthetic and natural fibres used in making hybrid composites are shown in Figure 1.

The bond strength between the matrix and the reinforcement is given by the interlaminar shear strength (ILSS), which gives the matrix/reinforcement performance. Shear, tensile and compressive forces are involved in interlaminar shear. Using the short beam specimen in the three-point bending method is the most commonly used method to determine the ILSS from all the various methods that have been reported to determine the ILSS [48]. The three-point bending method is widely used, as this method is simple to conduct and requires relatively fewer materials to determine the failure strength. These tests only provide an apparent shear strength, and the results obtained from these tests can be used as a quality control measure [47,48]. Matrix, reinforcement, fibre volume fraction, cracks and voids are some of the main properties which help in predicting the ILSS of a composite material.

Ease of handling during processing, carbon-neutral nature, cost-effectiveness, eco-friendliness, biodegradability, excellent mechanical properties and higher specific strength are some of the reasons for the extensive studies that have been conducted on flax-based composites. Composites made from flax fibres and other natural fibres have been used in various applications like automotive [49,50,51], biomedical prosthetic devices [52,53,54], wind turbine blades [55,56], construction [57,58,59,60,61], printed circuit boards [62], aerospace [63,64,65,66] and marine [67,68].

There is limited study conducted on ILSS of hybrid composites using natural fibres, e.g., flax, and high-performance fibres, e.g., glass. The current research dealt with hybrid polymer composites developed from sandwiched reinforcements using flax and glass woven fabrics. The interlaminar shear strength (ILSS) of the developed composites was studied using different combinations and arrangements of the glass and flax fabrics. Finite element analysis based on ANSYS was used to determine the ILSS for the hybrid composites. Further, experimental testing of the ILSS was carried out in order to validate the predicted performance. The comparison of simulated values with the tested values was conducted. Microscopic analysis was conducted to study the fracture in between the layers and interfacial debonding between the fibre and the matrix. The findings are important for understanding the performance of hybrid composites in real loading conditions in automotive frames and other components.

## 2. Materials and Methods

### 2.1. Materials

Given below in Table 1 are the properties of the materials used in the preparation of the composites.

High-performance fabrics were used as outer layers in various other studies [41,42,43], but in this study flax fabric has been used as the outer layer.

### 2.2. Methods

#### 2.2.1. Modelling Software

Using Solidworks 2024 a 3-D model was generated of the structure. Creep, plasticity, large deflection, stress stiffening, large strain capabilities and swelling were assumed for each element in the model, and eight nodes, each with three degrees of freedom, defined an element.

For the purposes of simulating the model, ANSYS 2024 was used, and the mechanical performances were predicted. The yarn/tow models were considered as a single element instead of individual filaments to improve the efficiency of simulation, as considering multiple filament models would lead to the increase in the nodes and elements, which would again lead to the increase in simulation times. The three-dimensional model was generated in Solidworks, with the following dimensions:

Length: 15 mm

Width: 5 mm

Height: 2 mm

#### 2.2.2. Sample Preparation

**Figure 2:**
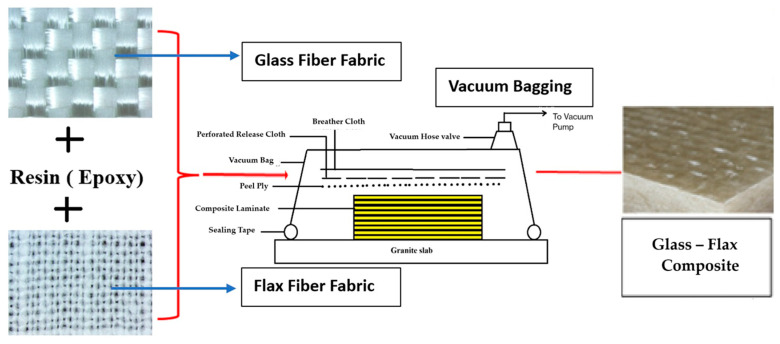
Flowchart representing the production of composites.

Using the hand layup method, the samples of the composites were made, which was then followed by the vacuum bagging process. Figure 2 shows the flowchart representing the production of composites.

The steps followed in the preparation of the samples are given below:

1. Prior to the application of the first layer of fabric, a releasing agent is to be applied onto the surface.
2. The subsequent layers are added to the base fabric by coating the base fabric with epoxy resin and placing the next layer of fabric. All the samples had a fibre volume fraction of 45%.
3. To separate the samples from the moulds easily, auxiliary fabric was used.
4. A pressure of 1000 mbar was applied for 2 h and the samples were allowed to be cured at room temperature for 24 h and subsequently the samples were cured at 120 °C for 2 h.

The samples produced had a thickness (2 ± 0.1 mm) and a volume fraction of (0.45), each with 8 layers of fabrics. From the samples produced, a total of 48 samples were selected which had both pure glass fabric composites and pure flax fabric composites, along with samples with a combination of different layers of glass fabrics and flax fabrics.

The epoxy resin used was a two-component structural epoxy resin LH 288 with hardener H 282 from Havel Composites CZ s. r. o., Prague, Czech Republic, which was used as the resin. This matrix is characterized by a low viscosity of 500–900 mPa × s at 25 °C, which is important for ensuring sufficient wetting of fabrics by lamination. The density is 1100–1200 kg/m^3^ at 25 °C. The volume fraction of matrix used for impregnation was 0.55.

Table 2 shows the samples which were prepared for testing:

### 2.3. Experimental Testing

#### 2.3.1. Interlaminar Shear Testing

According to ASTMD 2344 [71], the samples were cut, and the ILSS test was conducted on a Zwick Roell tensile testing machine (Ulm, Germany) with a 100 kN load cell. The test speed was set at 1 mm/min, and the ILSS was determined for all the samples, and the parameters of the samples used are as follows:

Length of sample: 15 mm

Breadth of sample: 5 mm

Height of sample: 2 mm

**Figure 3:**
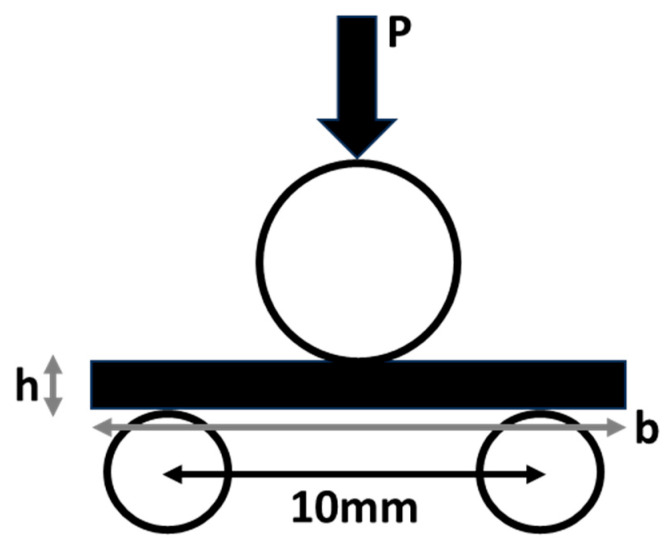
Schematic of ILSS test.

Equation (1) given below gives the ILSS:

(1) $$\mathrm{ILSS}=\frac{0.75\;\times \;F_{m}}{b\;\times \;h}$$

where, ILSS is interlaminar shear strength in MPa, *F_m_* is the maximum load at failure in N, *b* is the specimen width in mm and *h* is the specimen height in mm as shown in Figure 3. All the experiments were conducted under ambient conditions, i.e., 25 ± 2 °C and 65% relative humidity (RH).

#### 2.3.2. Fractured Surface Morphology

In order to understand the matrix and reinforcement materials failures in the composite, the morphology of the fractured sample was studied. The investigation was conducted by capturing high-resolution images using a Field Emission Scanning Electron Microscope (Karl Zeiss, Geneva, Switzerland). The images were captured at various levels of magnification. Since the samples used in the ILSS test were small, the whole sample was used to capture the morphology of the fractures.

### 2.4. Modelling Methodology

**Figure 4:**
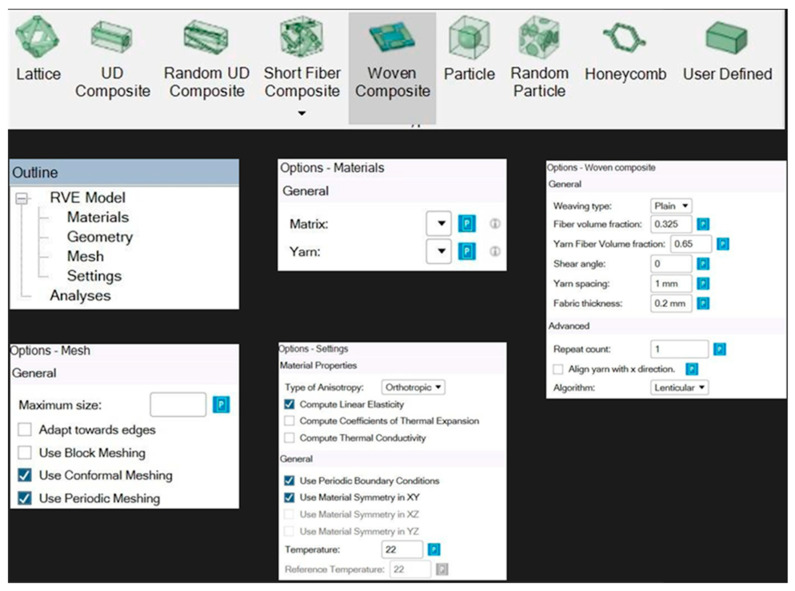
Parameters chosen to create a woven fabric.

**Figure 5:**
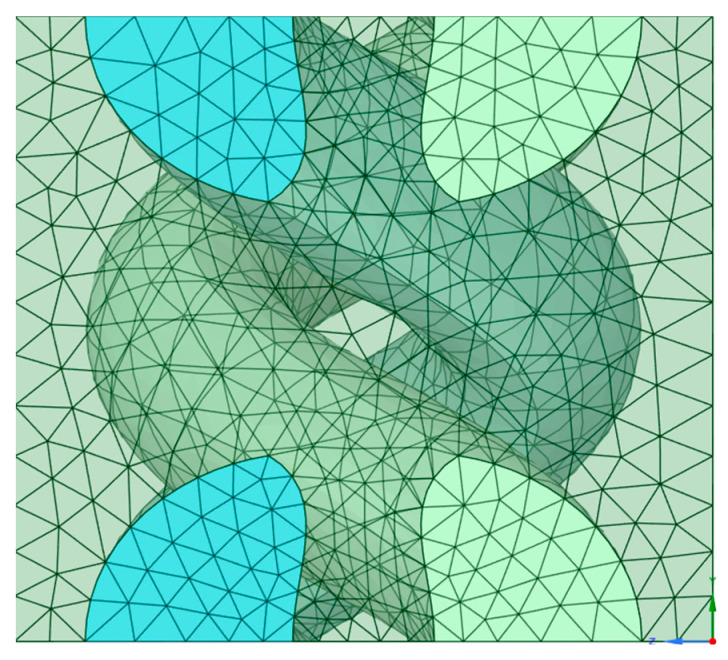
Basic repeating unit of the woven fabric in Space Claim of the Material Designer Module.

A woven fabric was created in the material designer module of ANSYS, and the same was used accordingly to create the different layers in the stacking for modelling and testing in ANSYS. In the material designer module in ANSYS, the woven fabric option was chosen, and the various details relating to that of the woven fabric, such as yarn volume, yarn spacing, shear angle, fabric thickness and yarn volume fraction, were entered, and the matrix and type of yarn were also defined. Later, the orthotropic nature and the meshing parameters of the model were selected. Figure 4 gives a detailed representation of the parameters that were chosen while creating a woven fabric in the material designer module, and Figure 5 shows the basic repeating unit of the woven fabric with the meshing pattern.

As depicted in Figure 5, the bent geometry of the constituent yarns/tows is taken into consideration in the model unit cell. Further, the mechanical properties of the constituent fabric, which is actually the interlaced assembly of the crimped/bent fibres, used as input for the model. Therefore, the yarn deformation, interlacement, and inter-yarn friction are already included in the model.

Further, the fibre-matrix interface (Glass-Epoxy or Flax-Epoxy bonds) is also one of the components included while predicting the overall performance of the composites.

The final composite block, which is a rectangular block, is shown only for demonstration purposes.

**Figure 6:**
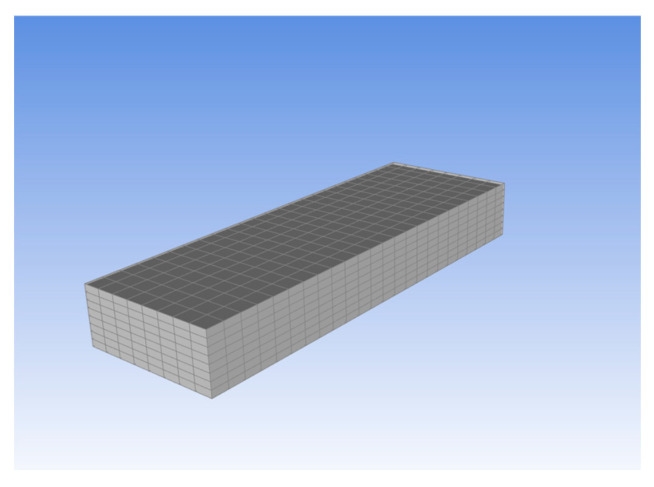
Sample with layers of fabric generated in the setup segment of the ACP (Pre) module.

The sample with layers of fabric generated in the setup segment of the ACP (Pre) module is shown in Figure 6.

The steps followed in creating all the samples in ANSYS are:

- The fabrics used to model the composite are modelled in the material designer module. In order to model the fabric, the following details are to be entered:

- Type of weave
- Spacing of the yarn
- Volume fraction of the fibre
- Thickness

b. After feeding the required data, the fabric is generated, and along with the fabric, data about the fabric is also generated.
c. The designer module generates data for the fabric, and this data is then transferred to the engineering data segment of the ACP (Pre) i.e., (Ansys Composite PrePost) module.
d. In the ACP (Pre) module the geometry that was created in the AUTOCAD software version 2024 (LIBTEX, Liberec, Czech Republic) as shown in Figure 6, is imported. The imported model is fed into the Geometry segment of the module.
e. The geometry which has been imported is now transferred to the Model segment of the ACP (Pre) module, and in this segment the imported geometry is designated as a flexible object, and the mesh for the sample is generated.
f. In the setup segment of the ACP (Pre) module, the fabrics along with their properties are created and applied to the model of the sample as shown in Figure 6. The fabrics that were created were 0.25 mm each to give a total thickness of 2 mm (thickness of the sample).
g. In the setup segment of the ACP (Pre), plies are generated with the appropriate properties.
h. The data generated in the setup segment is now transferred to the model segment in the Static Structural module.
i. In the Static Structural module, the boundary conditions are defined, the simulations are run, and the data is collected.

**Figure 7:**
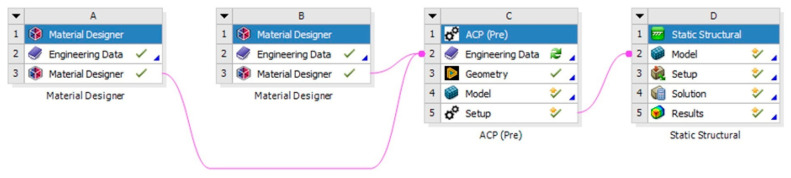
Project schematic, (**A**) Fabric material, (**B**), Matrix material, (**C**) Geometrical details, (**D**) Model set up.

Figure 7 shows the overall project schematic.

### 2.5. X-Ray 3-D Imaging

**Figure 8:**
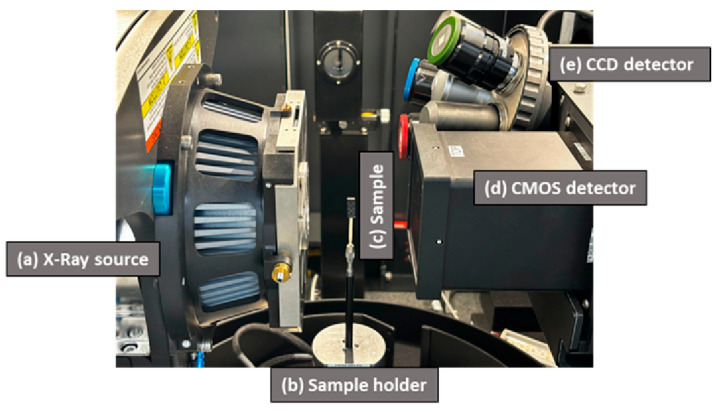
Zeiss Xradia versa 515 imaging system with sub-micron resolution used in the present study. The system consists of (**a**) an X-ray source [Polychromatic source with 160 kV tube voltage and 10 W power output], (**b**) a rotating sample holder, (**c**) a sample mounted on the sample holder, (**d**) a CMOS Flat Panel Detector- ideal for large field-of-view imaging and (**e**) a CCD Detector: having magnification options of 0.4×, 4×, 20×, and 40× for high-resolution studies across scales.

The Zeiss Xradia 515 versa, a high-resolution Xray Microscope (XRM), (Karl Zeiss, Geneva, Switzerland) was used in the present study to generate the 3-D images of the same specimen before and after the interlaminar shear test. A specimen mounted in the XRM is shown in Figure 8. The important features of the XRM used in the present are (a) a small spot-size stable X-ray source (30–160 kV, maximum 10 W), (b) an ultra-high precision 4-degrees-of-freedom sample stage (X, Y, Z and 360° rotation), (c) a dual-stage detector system with a detector turret of multiple objectives at different magnifications with optimised scintillators, and (d) a powerful software program with scout-and-scan acquisition and reconstruction capability. The settings used for 3-D X-ray microscopy analysis of the composite specimen before and after the ILSS test are given in Table 3.

## 3. Results and Discussion

### 3.1. Experimental Results

**Figure 9:**
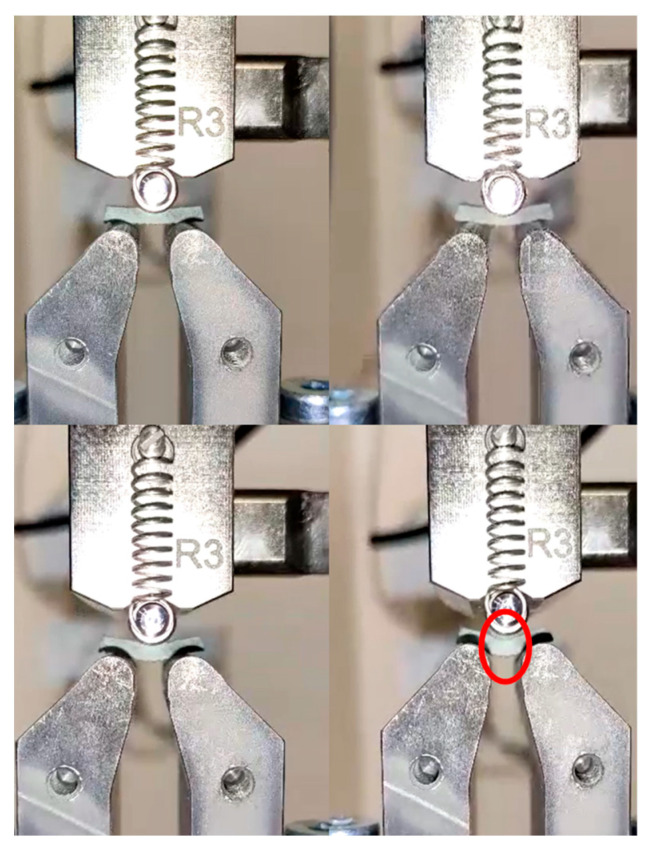
Series of images showing the ILSS testing of a sample shown in the red circle.

**Figure 10:**
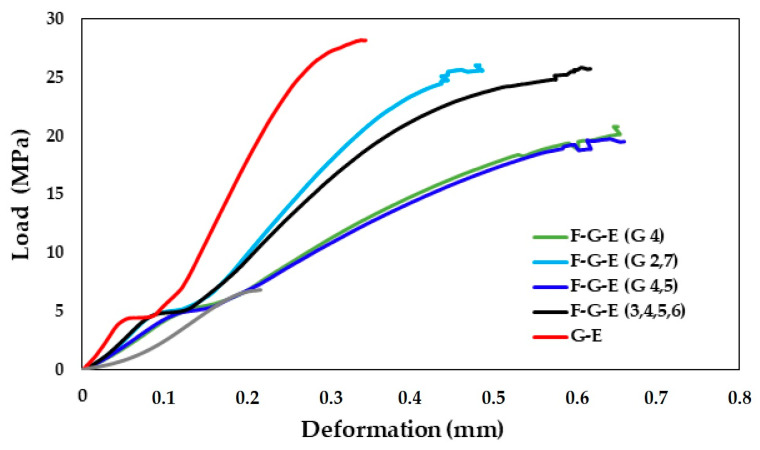
Load—Deformation curves for the samples.

**Figure 11:**
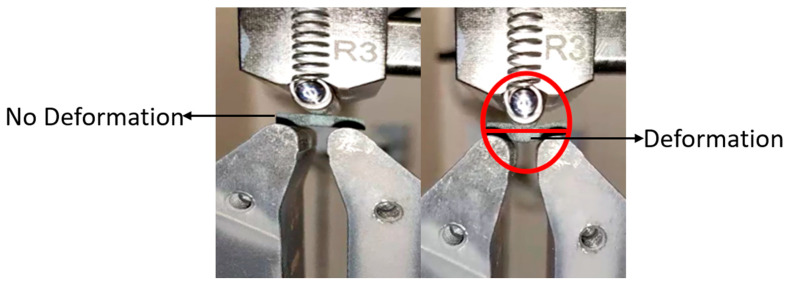
Before and after the application of load on a sample. The deformation is shown inside the red circle.

A total of 48 samples were tested according to ASTMD 2344 standards. In Figure 9 there are a series of images showing the ILSS testing of a sample which culminate with an image of the sample with a large deformation which has been highlighted. The following results were obtained as shown in Table 4, and the load—deformation curves for the samples derived from the results are given in Figure 10 and Figure 11, which show a sample before and after the application of load with the deformation.

The load-deformation curves for pure and hybrid composite specimens plotted in Figure 10 do not display linear behaviour till the failure, and this is a common observation in the short beam shear-based interlaminar shear strength tests. The plotted curves rather display the combined presence of pure shear mode in the initial stage and bending mode in the later part, with the implication that the initial part of the curve is considered close to the pure shear mode [72]. This behaviour was seen with the pure and the hybrid composites considered in the present study, may be due to the earlier onset of the bending mode, and is an indicator of the quality of the interface between the reinforcement and the matrix leading to delamination. These findings are in line with the reported findings on hybrid composites involving glass, hemp and basalt fibres [73]. Various aspects of the ILSS tests conducted are discussed in detail in the ensuing paragraphs.

From the obtained results, it can be observed that F-E (Flax-Epoxy) composite has lower interlaminar shear strength when compared to the other composite samples. The interlaminar shear strength of the G-E (Glass-Epoxy) sample is greater than the other samples, but its interlaminar shear strength is almost equal to that of the composite sample, which has 4 layers of glass fabric composite. It can also be noted from the results that the interlaminar shear strength of the samples increases with the increase in the number of glass layers; this is similar to the reported results where the ILSS of the flax composites improves with the inclusion of glass fibre layers in the hybrid composite [74].

### 3.2. Effect of Stacking Sequence on ILSS

Apart from the pure flax F-E and glass G-E composites, the present study considered four varieties of hybrid composites of flax and glass fibres wherein the glass layer/s (a) form the core, as in F-G-E (G 4,5) with two layers and F-G-E (G 3,4,5,6) with four layers; (b) form the penultimate layers-2nd and 7th layers, as in F-G-E (G 2,7); and (c) form an asymmetric core- 5th layer from the top, as in F-G-E (G 4). Unlike many of the reported studies with glass or any other high-performance fabric as outer layers [37,38,39,40,41], the present study differs in making the glass layers as penultimate ones to investigate the interlaminar shear properties. Prior to the study, it was hypothesised that (a) pure glass composite will have the highest shear strength commensurate with the number of glass layers and (b) the interlaminar shear strength will increase with the increase in the number of layers of glass in the hybrid composites irrespective of their position in the stack.

### 3.3. Simulation of Samples

**Figure 12:**
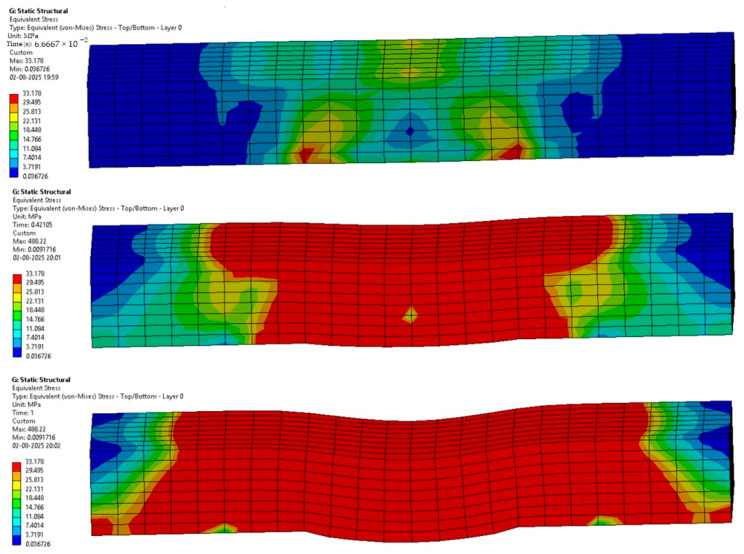
Phases in the simulation of Sample 1: Flax-Epoxy (F-E).

**Figure 13:**
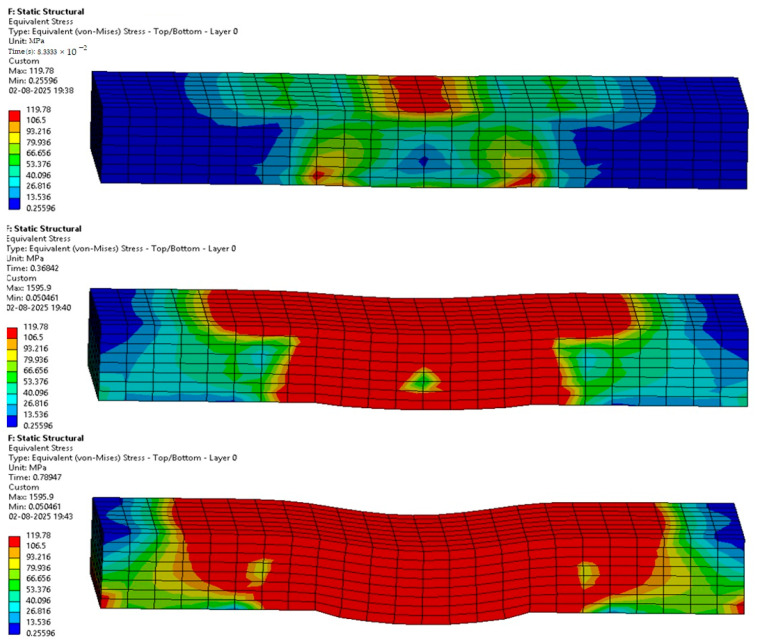
Phases in the simulation of Sample 2: Glass-Epoxy (G-E).

**Figure 14:**
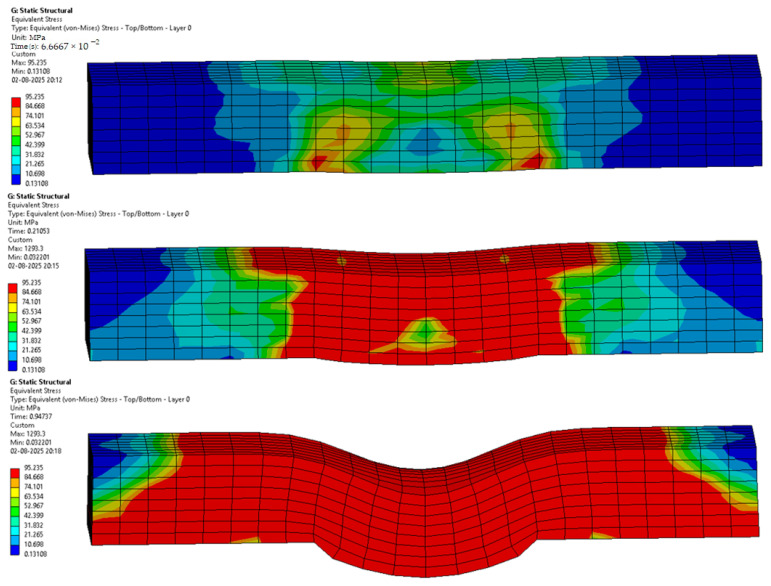
Phases in the simulation of Sample 3: Flax-Glass-Epoxy (F-G-E) (G 4).

**Figure 15:**
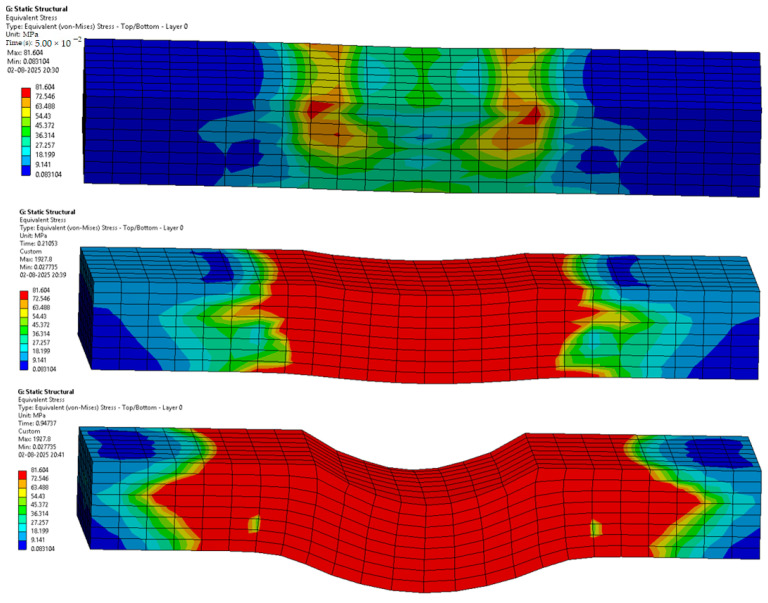
Phases in the simulation of Sample 4: Flax-Glass-Epoxy (F-G-E) (G 4,5).

**Figure 16:**
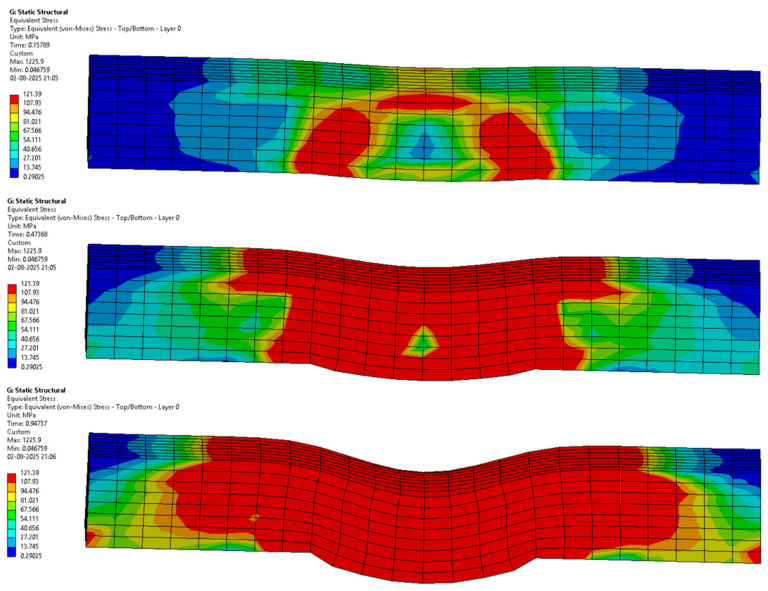
Phases in the simulation of Sample 5: Flax-Glass-Epoxy (F-G-E) (G 2,7).

**Figure 17:**
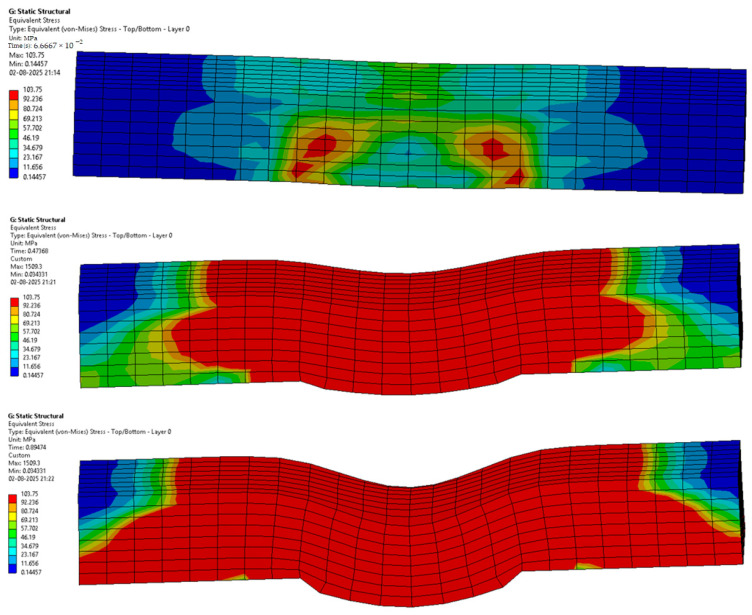
Phases in the simulation of Sample 6: Flax-Glass-Epoxy (F-G-E) (G 3,4,5,6).

The following Figure 12, Figure 13, Figure 14, Figure 15, Figure 16 and Figure 17 represent the simulation of the samples.

Sample 1: Flax-Epoxy (F-E).

Figure 12 represents a sequence of images which gives the phases in the testing of sample 1: Flax-Epoxy (F-E). Where it can be seen that in the initial stages the deformation is very less, and in the final stage, we can see that the deformation of sample 1: F-E is the greatest, resulting in the formation of cracks in the sample.

From the variation in the colour patterns in the elements in the sequence of images, it can be clearly seen where the breakage can occur in the final stage of the sequence of images.

Sample 2: Glass-Epoxy (G-E).

Figure 13 represents a sequence of images which gives the stages in the testing of sample 2: Glass-Epoxy (G-E). Where it can be seen that in the initial phases the deformation is very less, and in the final stage, we can see that the deformation of sample 2: G-E is the greatest, resulting in the formation of cracks in the sample.

From the variations in the colour patterns in the elements in the sequence of images, it can be clearly seen where the breakage can occur in the final stage of the sequence of images.

Sample 3: Flax-Glass-Epoxy (F-G-E) (G 4).

Figure 14 represents a sequence of images which gives the stages in the testing of sample 3: Flax-Glass-Epoxy (F-G-E) (G 4). Where it can be seen that in the initial phases the deformation is much less, and in the final stage, we can see that the deformation of sample 3: F-G-E (G 4) is the greatest, resulting in the formation of cracks in the sample. From the variations in the colour patterns in the elements in the sequence of images, it can be clearly seen where the breakage can occur in the final stage of the sequence of images.

Sample 4: Flax-Glass-Epoxy (F-G-E) (G 4,5).

Figure 15 represents a sequence of images which gives the stages in the testing of sample 4: Flax-Glass-Epoxy (F-G-E) (G 4,5). Where it can be seen that in the initial phases the deformation is much less, and in the final stage, we can see that the deformation of sample 4: F-G-E (G 4,5) is the greatest, resulting in the formation of cracks in the sample. From the variations in the colour patterns in the elements in the sequence of images, it can be clearly seen where the breakage can occur in the final stage of the sequence of images.

Sample 5: Flax-Glass-Epoxy (F-G-E) (G 2,7).

Figure 16 represents a sequence of images which gives the phases in the testing of sample 5: Flax-Glass-Epoxy (F-G-E) (G 2,7). Where it can be seen that in the initial stages the deformation is very less, and in the final stage, we can see that the deformation of sample 5: F-G-E (G 2,7) is the greatest, resulting in the formation of cracks in the sample.

From the variations in the colour patterns in the elements in the sequence of images, it can be clearly seen where the breakage can occur in the final stage of the sequence of images.

Sample 6: Flax-Glass-Epoxy (F-G-E) (G 3,4,5,6).

**Figure 18:**
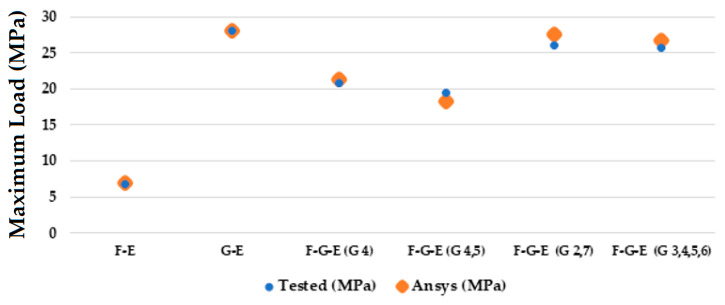
Comparing the maximum loads during experimental testing vs. maximum loads when running the simulations for the samples using Ansys.

Figure 17 represents a sequence of images which gives the phases in the testing of sample 6: Flax-Glass-Epoxy (F-G-E) (G 3,4,5,6). Where it can be seen that in the initial stages the deformation is very less, and in the final stage, we can see that the deformation of the sample 6: F-G-E (G 3,4,5,6) is the greatest, resulting in the formation of cracks in the sample. From the variations in the colour patterns in the elements in the sequence of images, it can be clearly seen where the breakage can occur in the final stage of the sequence of images. The results obtained after simulation of the samples were tabulated and compared with the results obtained from testing, which are given in Table 5 and Figure 18.

Upon comparing the results of the simulations with the tested results, from Figure 18 and Table 5 we can see that the values of interlaminar shear strength are very similar, and also from Table 5 we can see that the percentage error between the tested and simulated values ranges from a high of 6.25% to a low of 0.106%.

### 3.4. Failure Modes on ILSS Testing

The ASTM method followed in the present study employs short beam testing to measure the interlaminar shear strength. The method employs three-point bending, as shown in Figure 3, of a short composite specimen to produce failure in the shear mode while reducing the effect of bending stresses. While the shear stresses are independent of the support span length, the bending stresses vary linearly. To maintain a shear mode of failure and to reduce the effects of bending stresses, the standard lays down the dimensions of the specimen to be used in the testing. As there is no region of pure shear in three-point bending [75,76], the specimen during the loading may fail in different modes. The specimen, thus, potentially can exhibit the apparent interlaminar shear failure along with (a) compressive failure, (b) tensile failure or a combination of all three. While the interlaminar shear failure depends on the bonding between the matrix and reinforcement, the compressive and tensile failure depend on the mechanical properties of the fibre/s used as reinforcement. The method employed measures the ‘apparent’ interlaminar shear strength to compare the different materials under the same conditions of testing for quality assurance purposes [77,78].

The standard ASTM D2344 [71] employed in the current study provides a list of typical failure modes. Several researchers also demonstrated typical failure modes that the specimen may suffer on the interlaminar shear test. The standard also mentions that the typical failures may be preceded by local damage modes, such as trans-ply cracking, that are less obvious.

**Figure 19:**
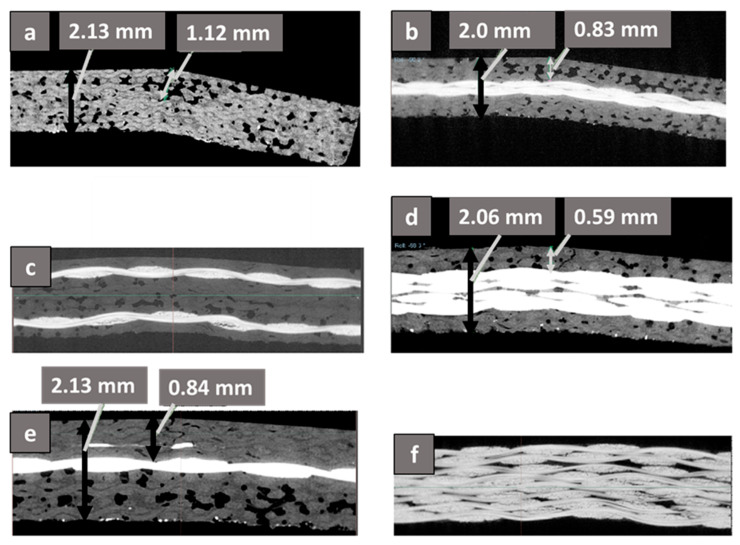
XRM of the hybrid composites in YZ plane showing the crack in (**a**) F-E, (**b**) F-G-E (G 4,5), (**c**) F-G-E (G 2,7). (**d**) F-G-E (G 3,4,5,6), (**e**) F-G-E (G 4) and (**f**) G-E.

The failure mode was investigated using the XRM technique, and Figure 19 shows the scanned post-test images in the YZ plane of the pure and the hybrid composites used in the present study. The YZ plane (sagittal plane bound by height and depth) was selected, as the scan reveals the depth of the crack that is suffered by the composite specimen in the interlaminar shear test. The XRM scan clearly highlights the presence of all the glass layers in G-E and glass layers in their designated positions in the hybrid composites and the absence of the glass layer in the pure flax F-E composite. The appearance of the glass as the bright layer is due to its high density as compared to the matrix and the reinforcement. The scans shown in Figure 19 reveal a combination of typical failures as specified in ASTM D2344. While the pure Flax-Epoxy composite (F-E) and hybrid composites, e.g., Flax-Glass-Epoxy (F-G-E) (G 4), (G 4,5), and (G 3,4,5,6), exhibited a combination of interlaminar shear and tensile failure with a crack on the entire width of the sample, pure glass G-E and hybrid specimen F-G-E (G 2,7) exhibited pure interlaminar shear and tensile/compressive failure with micro-damage and a perceptible bend at the centre but with no visible signs of cracks on the compressive or the tensile side, as revealed by the XRM scan. The noted behaviour of G-E and F-G-E (G 2,7) may be attributed to the better interface between the matrix and the reinforcement. Though we expected that the outer layer of the flax in F-G-E (G 2,7) would crack on the tensile side as in the other hybrid specimen, no crack was observed, and this may be attributed to the effective load transfer to the underlying glass layer through the matrix.

To analyse the effect of the number of glass layers on the depth of the crack, the reported depth of the crack was normalised with respect to the thickness of the specimen, and the normalised values are tabulated in Table 6. Pure flax F-E specimen with a normalised value of 0.526, obviously, exhibited a deep tensile crack as seen in Figure 19a that travelled almost to the neutral plane where the compressive and the tensile loads even out. The hybrid with the maximum number of glass layers in F-G-E (G 3,4,5,6) with a normalised value of 0.286 exhibits the least depth of tensile crack that stops at the glass layer, as seen in Figure 19b. The specimens of F-G-E (G 4,5) and F-G-E (G 4) have almost similar normalised values of 0.415 and 0.394, respectively, though it was expected that F-G-E (G 4,5) with two layers of glass would have a lower normalised value as compared to the F-G-E (G 4) specimen.

### 3.5. Morphology

The morphology of the fractured surface was investigated by using a Field Emission Scanning Electron Microscope (FESEM)-Gemini SEM 300 made by (Karl Zeiss, Geveva, Switzerland). In the present study, as shown in Table 6, the pure Glass-Epoxy composite (G-E) and Flax-Glass-Epoxy (F-G-E) composites (G 3,4,5,6) and (G 2,7) specimens suffer micro-damages on the tensile side, while all other specimens, e.g., F-E, F-G-E (G 4), F-G-E (G 4,5), and F-G-E (G 3,4,5,6), suffered extensive damage on the tensile side, leading to the formation of a crack over the entire width. Thus, we focused on the morphology study of the central portion of the specimen that experienced tensile force in the interlaminar shear test. Except for the pure G-E specimen, all the others have flax as outer layers, and we expected the failure mechanism of the flax fibre in the composite to be similar to that of a flax fibre subjected to tensile loading.

**Figure 20:**
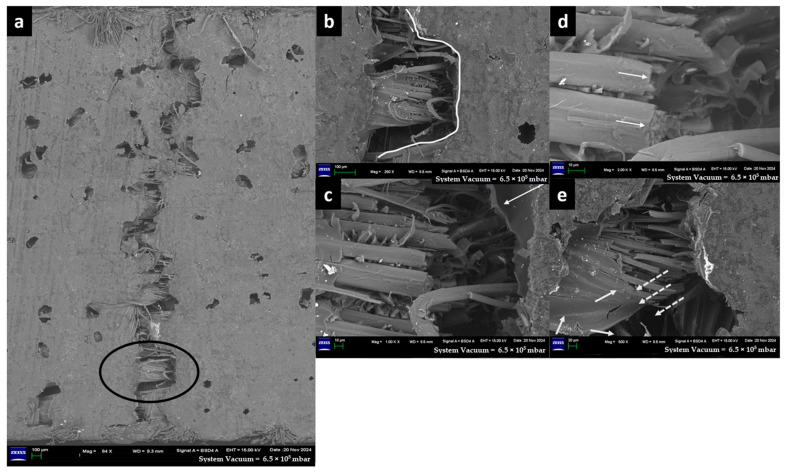
FESEM morphology of the fractured Flax-Epoxy (F-E) composite sample 1: Flax-Epoxy (F-E), (**a**) 84×, (**b**) 250×, (**c**) 1000×, (**d**) 2000×, (**e**) 500×. The black circle shows fractured tow, the white line shows the fracture of matrix and the white arrows show the fibre fractures.

**Figure 21:**
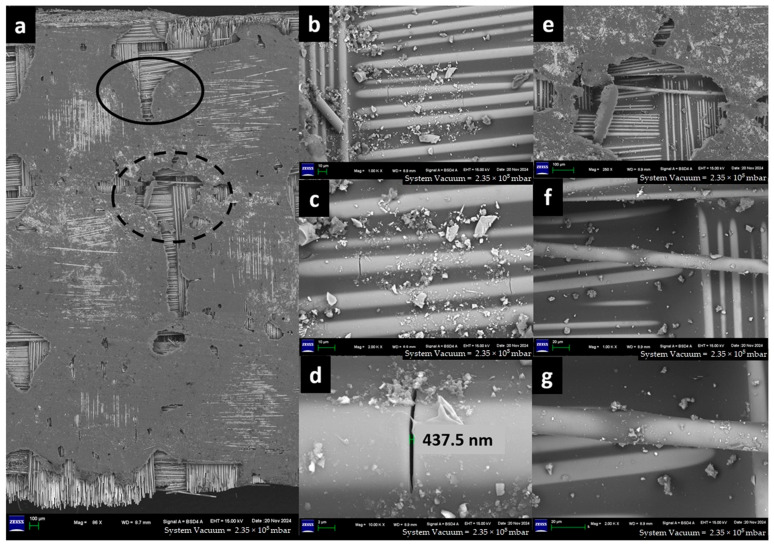
FESEM morphology of the fractured Flax-Epoxy (F-E) composite sample 2: Glass-Epoxy (G-E), (**a**) 86×, (**b**) 1000×, (**c**) 2000× lengthwise, (**d**) 10,000×, (**e**) 250×, (**f**) 1000×, (**g**) 2000× widthwise. The black circle shows fractured tow, dotted black circle shows delamination.

**Figure 22:**
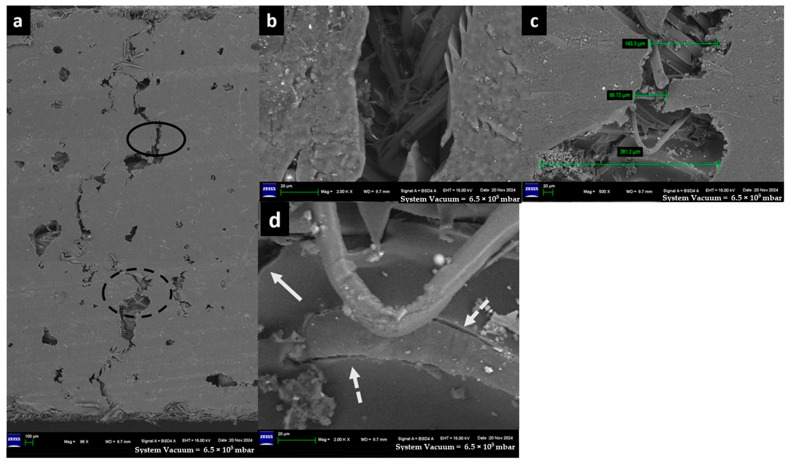
FESEM morphology of the fractured Flax-Epoxy (F-E) composite sample 3: Flax-Glass-Epoxy (F-G-E) (G 4), (**a**) 86×, (**b**) 2000× matrix, (**c**) 500×, (**d**) 2000× fibre. The black circle shows fractured tow, dotted black circle shows delamination, the white arrows show the fibre fracture and the green line shows the fracture width.

**Figure 23:**
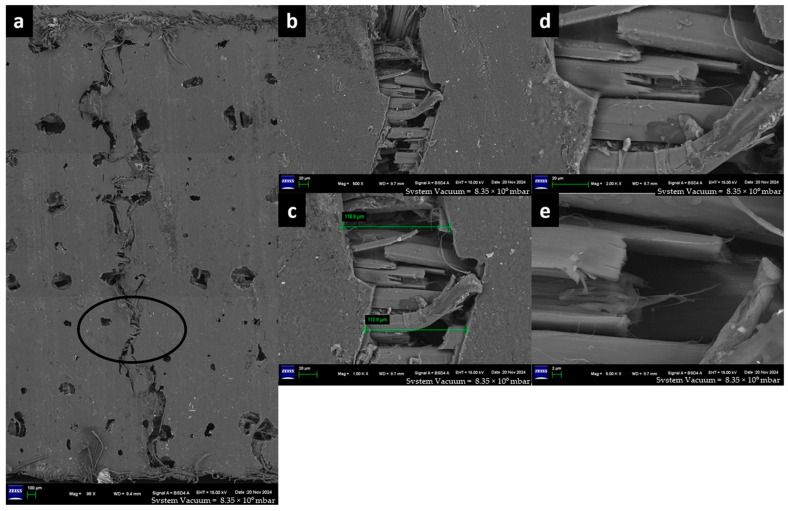
FESEM morphology of the fractured Flax-Epoxy (F-E) composite sample 4: Flax-Glass-Epoxy (F-G-E) (G 4,5), (**a**) 86×, (**b**) 500×, (**c**) 1000×, (**d**) 2000×, (**e**) 5000×. The black circle shows fractured tow and the green line shows the fracture width.

**Figure 24:**
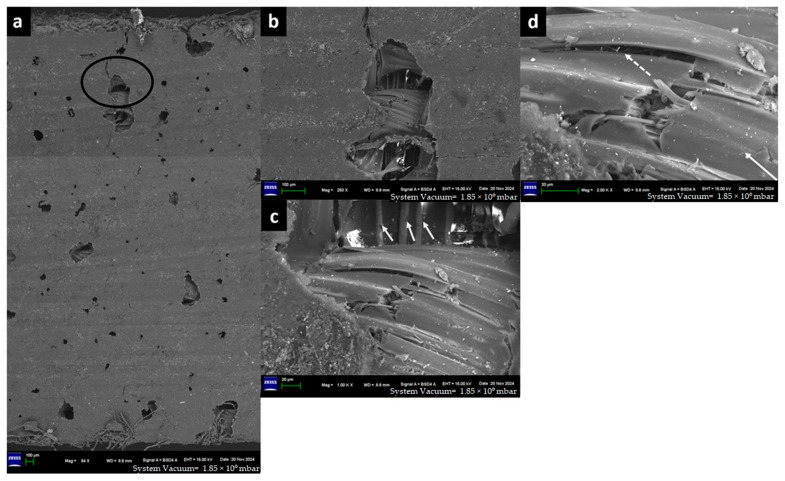
FESEM morphology of the fractured Flax-Epoxy (F-E) composite sample 5: Flax-Glass-Epoxy (F-G-E) (G 2,7), (**a**) 84×, (**b**) 250×, (**c**) 1000×, (**d**) 2000×. The black circle shows fractured tow and the white arrows show the fibre fracture.

**Figure 25:**
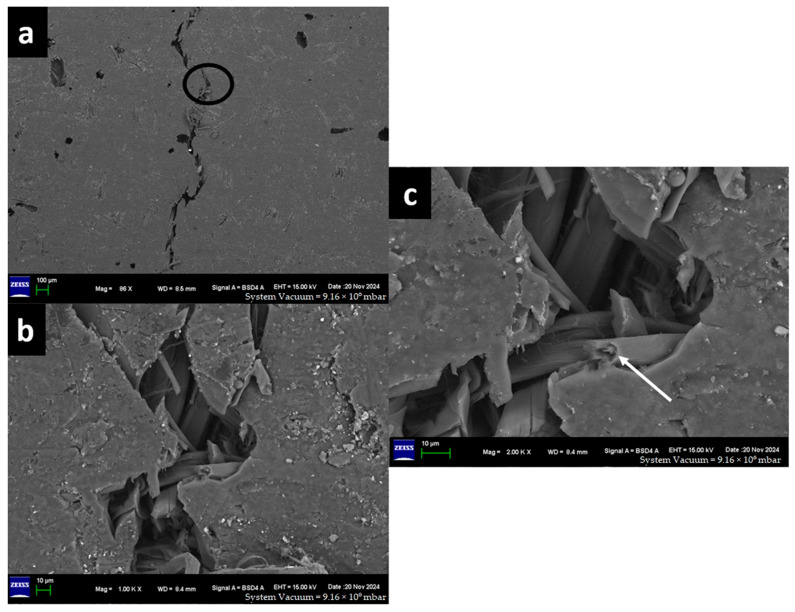
FESEM morphology of the fractured Flax-Epoxy (F-E) composite sample 6: Flax-Glass-Epoxy (F-G-E) (G 3,4,5,6), (**a**) 86×, (**b**) 1000×, (**c**) 2000×. The black circle shows fractured tow and the white arrow shows the fibre fracture.

The captured high-resolution surface images are shown in Figure 20 for F-E, Figure 21 for G-E, Figure 22 for F-G-E (G 4), Figure 23 for F-G-E (G 4,5), Figure 24 for F-G-E (G 2,7) and Figure 25 for F-G-E (G 3,4,5,6). Figure 20a -25a for all the composite samples represent the stitched-in/combined sections to show the presence of the crack throughout the width of the specimen. Although F-G-E (G 3,4,5,6) also showed the crack over its entire width, a middle part is shown in (a) of Figure 25. The stitched-in images shown in (a) of Figure 21 and Figure 24 for G-E and F-G-E (G 2,7), respectively, reveal the absence of the crack. As can be seen, the common failures of matrix and fibre, cracks in matrix, cracks in fibre, fibre pull-out and delamination are evident and are shown. Images for F-G-E (G 2,7) and G-E reveal interesting observations. Figure 24d of F-G-E (G 2,7) shows the initiation of the tensile failure of the flax fibre, and it is presumed that the obvious bridging provided by the underlying glass layer as seen in Figure 24c interrupts the completion of the tensile failure, which otherwise is seen in Figure 20c of F-E, Figure 23c of F-G-E (G 4,5), Figure 25c of F-G-E (G 3,4,5,6) and Figure 22c of F-G-E (G 4). This bridging effect has been discussed above and appears to be the major factor in realising shear strength value. This finding highlights the importance of having an underlying layer of glass fabric or any other high-performance fabric material in an application that demands the use of flax as an outer material, maybe to enhance the damping properties. Figure 21b–d of G-E show the initiation of the micro-damage in the form of crack/s on the surface of the glass fibre, marking the initiation of the process of tensile failure. The noted micro-damage appears to be localised, as we did not observe the cracks on the glass fibre even at higher magnification levels, as revealed by (e) at 250X, (f) at 1kX and (g) at 2kX of Figure 22 of G-E taken from a nearby location by zooming in on the broken elliptical portion.

Sample 1: Flax-Epoxy (F-E).

FESEM morphology of Flax-Epoxy (F-E) composite depicting (a) three combined sections to capture the entire crack on the ILSS test, (b) a zoomed-in elliptical portion of (a) to show the matrix failure (highlighted), (c) a zoomed-in portion of (b) to show the cracked matrix as indicated by an arrow, (d) a zoomed-in portion of (b) to show the broken reinforcement—flax fibre, indicating the brittle failure as indicated by the arrows and (e) cracks in the matrix—as indicated by solid arrows and delamination—as indicated by broken arrows.

Sample 2: Glass-Epoxy (G-E).

Field emission scanning electron microscopy (FESEM) of G-E composite depicting (a) three combined sections to capture the absence of cracks on the surface after the ILSS test but for the loss of the matrix randomly; (b), (c) and (d) depict zoomed-in solid elliptical portions to show the transverse crack of the glass fibre as pointed out by the arrows; (e), (f) and (g) depict zoomed-in broken elliptical portions to show the absence of the crack/s seen in (b), (c) and (d).

Sample 3: Flax-Glass-Epoxy (F-G-E) (G 4).

FESEM of F-G-E (G 4) composite: (a) three combined sections to capture the entire crack on the ILSS test, (b) zoomed-in solid elliptical portion of (a) to show the matrix failure and broken reinforcement- flax fibre of the hybrid. An arrow points to the transverse crack followed by longitudinal crack propagation, (c) a zoomed-in portion of the broken elliptical portion (a) to show the width of the cracked matrix and the broken reinforcement—Flax fibre of the hybrid, indicating the brittle failure as indicated by a solid arrow. The broken arrow suggests that the fibre displaying the brittle failure is pulled out of the matrix, and (d) is a zoomed-in portion of the bottom half of (c) to show the cracked matrix as indicated by a solid arrow and delamination as indicated by broken arrows.

Sample 4: Flax-Glass-Epoxy (F-G-E) (G 4,5).

FESEM of F-G-E (G 4,5) composite depicting (a) three combined sections to capture the entire crack on the ILSS test, (b) zoomed-in elliptical portion of (a) to show the matrix failure, (c) zoomed-in portion of (b) to show the width of the cracked matrix, (d) zoomed in portion of (c) to show the broken reinforcement –Flax fibre of the hybrid, indicating the transverse crack followed by longitudinal crack propagation and (e) zoomed-in portion of (d) to show the longitudinal crack.

Sample 5: Flax-Glass-Epoxy (F-G-E) (G 2,7).

FESEM of F-G-E (2,7) composite depicting (a) three combined sections to capture the crack seen only at the top on the ILSS test, and the solid arrows point to the cracks in the matrix, (b) the zoomed-in elliptical portion of (a) to show the matrix failure, and (c) the zoomed-in portion of (b) to show the cracked matrix and the flax fibre of the hybrid. Also, the arrows show the uncracked glass fibre and the obvious bridging effect due to the stacking of flax over the glass fabric, and (d) is a zoomed-in portion of (c) to show the broken reinforcement—flax fibre, indicating the transverse crack and thus the initiation of the tensile failure. Also, the solid arrow points to the crack in the matrix, and the broken arrow points to delamination.

Sample 6: Flax-Glass-Epoxy (F-G-E) (G 3,4,5,6).

FESEM of F-G-E (G 3,4,5,6) composite depicting (a) the middle section to capture the crack on the ILSS test, (b) the zoomed-in elliptical portion of (a) to show the matrix failure, and (c) the zoomed-in portion of (b) to show the cracked matrix and the broken reinforcement—flax fibre of the hybrid, indicating the transverse crack followed by longitudinal crack propagation as indicated by an arrow.

Figure 20c of F-E, Figure 23c of F-G-E (G 4,5), Figure 25c of F-G-E (G 3,4,5,6) and Figure 22c of F-G-E (G 4) show the tensile failure of flax fibre, while Figure 24c of F-G-E (G 2,7) shows the initiation of the tensile failure, and the mechanisms, as seen, include just the transverse and brittle failure (Figure 20d of F-E, Figure 22c of F-G-E(G 4)) or a transverse failure coupled with longitudinal splitting (Figure 23d of F-G-E (G 4,5), Figure 25c of F-G-E (G 3,4,5,6)) or a combination of transverse failure and longitudinal splitting. Also, the initiation of the tensile failure seen in Figure 24d of F-G-E (G 2,7) appears to be leading to transverse and brittle failure. These failure mechanisms for the flax fibre seen in the present study agree with the reported findings in literature [79,80,81].

## 4. Conclusions

The hybridisation approach is vigorously pursued by the research organisations, academia and the industry in an effort to reduce the threatening environmental issues associated with the production of high-performance synthetic fibres while promoting the optimal/limited use of natural fibres to develop appropriate composites for the semi-structural and the structural applications. Hybridisation of natural fibres with high-end carbon, glass, Kevlar, PEEK and others is imperative for the manufacture of hybrid composites with enhanced properties. The present study focussed on the study of the interlaminar shear strength of the pure and hybrid composites made of natural flax and synthetic glass fibre in a particular stacking sequence with flax forming the outer layers.

The main findings of the study are:

- Hybridisation of flax with glass fibre improves the interlaminar shear strength considerably as compared to that of the flax fibre alone, and the reported values are higher than that of the pure flax and lower than that of the pure glass fibre.
- Hybrid samples of F-G-E (G 2,7) with glass as the penultimate layers and F-G-E (G 3,4,5,6) with four layers of glass in the core have almost the same strength. When we compare these hybrid samples with the sample which has 8 layers of glass G-E, it is seen that the pure glass fibre sample is stronger.
- When comparing the two hybrid samples with two layers of glass, F-G-E (2,7) and F-G-E (G 4,5), it can be seen that F-G-E (G 4,5) has a lower strength than F-G-E (G 2,7), which can be attributed to a host of reasons like good fibre-matrix interface, low void content and bridging effect.
- The stated hypothesis at the start of the present study that the interlaminar shear strength will increase with the increase in the number of layers of glass in the hybrid composites irrespective of their position in the stack stands rejected, as the number and position of the glass layers in the stack had an overriding influence.
- Using the advanced XRM imaging technique, the depth of the crack on the tensile side of the specimen has been measured for the first time, to the best of the authors’ knowledge, to study the progression of the crack. XRM proves to be an excellent technique, as it does not involve any sample/section preparation.
- All the specimens considered in the present study, except G-E and F-G-E (G 2,7), failed primarily due to the combination of interlaminar shear and tensile failure as seen by a crack over the entire width as revealed by the XRM images. G-E and F-G-E (G2,7) specimens failed due to the interlaminar shear and the micro-damages suffered on the tensile and compressive sides.
- The observed tensile failure of the flax fibre was confirmed as the morphology exhibited the transverse and brittle failure or the transverse failure coupled with longitudinal splitting.

## Figures and Tables

**Table 1:** Properties of fibres and fabric [40,69,70,71,72].

Properties	Flax	Glass
Diameter of fibre (µm)	20 ± 1.2	21 ± 1.1
Linear density of fibre (Tex, g/km)	21 ± 1.2	20 ± 1.1
Linear density of yarn (Tex, g/km)	600 ± 11	600 ± 2
Density (g/cm^3^)	1.5 ± 0.1	2.48 ± 0.2
Areal density of Fabric (g/m^2^)	600 ± 10	600 ± 25
Young’s modulus of yarn (GPa)	86.5 ± 1.4	37.5 ± 0.8
Bulk modulus for yarn (GPa)	37.7 ± 1.5	15.4 ± 1.1
Warp density in fabric (cm^−1^)	15	15
Weft density in fabric (cm^−1^)	14	14
Strength (GPa)	2.47 ± 0.05	4.65 ± 0.15

**Table 2:** Samples prepared.

Samples	Sample Code	Orientation	Representative Sample	Layers	Fabrics							
					1	2	3	4	5	6	7	8
Flax- Epoxy	F-E	0/90	** 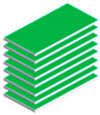 **	8	F	F	F	F	F	F	F	F
Glass-Epoxy	G-E	0/90	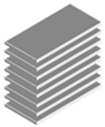	8	G	G	G	G	G	G	G	G
Flax-Glass-Epoxy (G 4)	F-G-E (G 4)	0/90	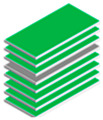	8	F	F	F	G	F	F	F	F
Flax-Glass-Epoxy (G 3,4)	F-G-E (G 4,5)	0/90	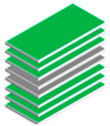	8	F	F	G	G	F	F	F	F
Flax-Glass-Epoxy (G 2,7)	F-G-E (G 2,7)	0/90	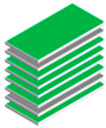	8	F	G	F	F	F	F	G	F
Flax-Glass-Epoxy (G 3,4,5,6)	F-G-E (G 3,4,5,6)	0/90	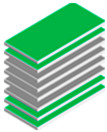	8	F	F	G	G	G	G	F	F

**Table 3:** Details of the machine settings used for scanning.

Machine Parameter	Setting
Accelerating Voltage (kV)	40
Power (W)	3
Exposure Time (s)	2.5
Voxel Volume (µm^3^)	14
X-ray Source to Sample distance (mm)	87
Sample to Detector distance (mm)	17
Filter	Air

**Table 4:** Experimental results.

Sample Codes	Load (MPa)	Deformation (mm)	Number of Samples Tested
F-E	6.87	0.22	8
G-E	28.13	0.35	8
F-G-E (G 4)	20.75	0.62	8
F-G-E (G 4,5)	19.55	0.66	8
F-G-E (G 2,7)	26.0018	0.48	8
F-G-E (G 3,4,5,6)	25.713	0.62	8

**Table 5:** Comparison of results obtained.

Sample Codes	Load (MPa)	Ansys (MPa)	Percentage Error (%)
F-E	6.87	7.01	2.03
G-E	28.13	28.1	0.106
F-G-E (G 4)	20.75	21.33	1.93
F-G-E (G 4,5)	19.55	18.28	6.25
F-G-E (G 2,7)	26.0018	27.5	5.76
F-G-E (G 3,4,5,6)	25.713	26.8	4.22

**Table 6:** Details of the typical failures exhibited by composite specimens after the interlaminar shear test.

Composite Specimen Codes	Type of Failure Noticed in the Sample	Depth of Crack, a (mm) in Relation to the Thickness, b (mm) of the Composite Specimen	Normalised Depth of Crack, a/b
F-E	Interlaminar shear with Tensile failure exhibiting a crack over the entire width of the sample	1.12/2.13	0.526
F-G-E (G 4,5)	Interlaminar shear with Tensile failure exhibiting a crack over the entire width of the sample	0.83/2.0	0.415
F-G-E (2,7)	Interlaminar shear with micro-damage on the tensile and compressive side but without the crack	--	--
F-G-E (G 3,4,5,6)	Interlaminar shear with Tensile failure exhibiting a crack over the entire width of the sample	0.59/2.06	0.286
F-G-E (G 4)	Interlaminar shear with Tensile failure exhibiting a crack over the entire width of the sample	0.84/2.13	0.394
G-E	Interlaminar shear with micro-damage on the tensile and compressive side but without the crack	--	--

## Data Availability

The original contributions presented in the study are included in the article. Further inquiries can be directed to the corresponding author(s).

## References

[1] Kumar S., Sharma N., Biswas R., Singh K.K. (2023). Effect of temperature on the flexural and ILSS behaviour of symmetric and asymmetric basalt fibre-reinforced polymer composites. Mater. Today Proc..

[2] Hawileh R.A., Abu-Obeidah A., JaAbdalla J.A., Al-Tamimi A. (2015). Temperature effect on the mechanical properties of carbon, glass and carbon–glass FRP laminates. Constr. Build. Mater..

[3] Kumar S., Singh K.K., Ramkumar J. (2020). Comparative study of the influence of graphene nanoplatelets filler on the mechanical and tribological behavior of glass fabric-reinforced epoxy composites. Polym. Compos..

[4] Zhou Y., Fan Z., Du J., Sui L., Xing F. (2015). Bond behavior of FRP-to-concrete interface under sulfate attack: An experimental study and modeling of bond degradation. Constr. Build. Mater..

[5] Akshat T., Petru M., Mishra R.K. (2025). Numerical Modelling of Hybrid Polymer Composite Frame for Selected Construction Parts and Experimental Validation of Mechanical Properties. Polymers.

[6] Realfonzo R., Martinelli E., Napoli A., Nunziata B. (2013). Experimental investigation of the mechanical connection between FRP laminates and concrete. Compos. Part B Eng..

[7] Wong P.M.H., Wang Y.C. (2007). An experimental study of pultruded glass fibre reinforced plastics channel columns at elevated temperatures. Compos. Struct..

[8] Huang X. (2009). Fabrication and Properties of Carbon Fibers. Materials.

[9] Vandeurzen P., Ivens J., Verpoest I. (1996). A three-dimensional micromechanical analysis of woven-fabric composites: I. Geometric analysis. Compos. Sci. Technol..

[10] Thwe M.M., Liao K. (2003). Durability of bamboo-glass fiber reinforced polymer matrix hybrid composites. Compos. Sci. Technol..

[11] Jacob M., Joseph S., Pothan L.A., Thomas S. (2005). A study of advances in characterization of interfaces and fiber surfaces in lignocellulosic fiber-reinforced composites. Compos. Interfaces.

[12] Arumugam V., Militky J., Davies L., Slater S. (2018). Thermal and water vapor transmission through porous warp knitted 3D spacer fabrics for car upholstery applications. J. Text. Inst..

[13] Burgani T., Alaie S., Tehrani M. (2022). Modeling Flexural Failure in Carbon-Fiber-Reinforced Polymer Composites. J. Compos. Sci..

[14] Behera B.K., Pattanayak A.K., Mishra R. (2008). Prediction of Fabric Drape Behaviour using Finite Element Method. J. Text. Eng..

[15] Tsai K.-H., Chiu C.-H., Wu T.-H. (2000). Fatigue behavior of 3D multi-layer angle interlock woven composite plates. Compos. Sci. Technol..

[16] Suriani M.J., Ilyas R.A., Zuhri M.Y.M., Khalina A., Sultan M.T.H., Sapuan S.M., Ruzaidi C.M., Wan F.N., Zulkifli F., Harussani M.M. (2021). Critical Review of Natural Fiber Reinforced Hybrid Composites: Processing, Properties, Applications and Cost. Polymers.

[17] Wagh J.P., Malagi R.R., Madgule M. (2024). Investigative studies on natural fiber reinforced composites for automotive bumper beam applications. J. Reinf. Plast. Compos..

[18] Bidadi J., Arabha M., Googarchin H.S. (2024). Adhesive bonding in automotive hybrid multi-cell square tubes: Experimental and numerical investigation on quasi-static axial crashworthiness performance. Int. J. Adhes. Adhes..

[19] Venkataraman M., Xiong X., Marek J., Yao J., Zhu G. (2018). Electrospun nanofibrous membranes embedded with aerogel for advanced thermal and transport properties. Polym. Adv. Technol..

[20] Chandan V., Mishra R.K., Kolar V., Muller M., Hrabe P. (2024). Green hybrid composites partially reinforced with flax woven fabric and coconut shell waste-based micro-fillers. Ind. Crops Prod..

[21] Bachtiar D., Sapuan S.M., Hamdan M.M. (2010). Flexural Properties of Alkaline Treated Sugar Palm Fibre Reinforced Epoxy Composites. Int. J. Automot. Mech. Eng..

[22] Nurazzi N.M., Asyraf M.R.M., Fatimah Athiyah S., Shazleen S.S., Rafiqah S.A., Harussani M.M., Kamarudin S.H., Razman M.R., Rahmah M., Zainudin E.S. (2021). A Review on Mechanical Performance of Hybrid Natural Fiber Polymer Composites for Structural Applications. Polymers.

[23] Lee C.H., Khalina A., Nurazzi N.M., Norli A., Harussani M.M., Rafiqah S.A., Aisyah H.A., Ramli N. (2021). The Challenges and Future Perspective of Woven Kenaf Reinforcement in Thermoset Polymer Composites in Malaysia: A Review. Polymers.

[24] Bidadi J., Hampaiyan M.H., Saeidi G.H., Akhavan-Safar A., da Silva L.F.M. (2024). Experimental and numerical investigation on the crashworthiness performance of double hat-section Al-CFRP beam subjected to quasi-static bending test. Polym. Compos..

[25] Bidadi J., Saeidi Googarchin H., Akhavan-Safar A., Carbas R.J.C., da Silva L.F.M. (2023). Characterization of Bending Strength in Similar and Dissimilar Carbon-Fiber-Reinforced Polymer/Aluminum Single-Lap Adhesive Joints. Appl. Sci..

[26] Suriani M.J., Zainudin H.A., Ilyas R.A., Petrů M., Sapuan S.M., Ruzaidi C.M., Mustapha R. (2021). Kenaf Fiber/Pet Yarn Reinforced Epoxy Hybrid Polymer Composites: Morphological, Tensile, and Flammability Properties. Polymers.

[27] Kalaprasad G., Thomas S., Pavithran C., Neelakantan N.R., Balakrishnan S. (1996). Hybrid Effect in the Mechanical Properties of Short Sisal/Glass Hybrid Fiber Reinforced Low Density Polyethylene Composites. J. Reinf. Plast. Compos..

[28] Rudov-Clark S., Mouritz A.P. (2008). Tensile fatigue properties of a 3D orthogonal woven composite. Compos. Part A Appl. Sci. Manuf..

[29] Wang Y., Hu S., Sun X. (2022). Experimental investigation on the elastic modulus and fracture properties of basalt fiber–reinforced fly ash geopolymer concrete. Constr. Build. Mater..

[30] Xie H., Yang L., Zhang Q., Huang C., Chen M., Zhao K. (2022). Research on energy dissipation and damage evolution of dynamic splitting failure of basalt fiber reinforced concrete. Constr. Build. Mater..

[31] Hassan T., Jamshaid H., Khan M.Q., Tichy M., Muller M. (2021). Factors Affecting Acoustic Properties of Natural-Fiber-Based Materials and Composites: A Review. Textiles.

[32] Wang A., Wang X., Xian G. (2020). Mechanical, Low-Velocity Impact, and Hydrothermal Aging Properties of Flax/Carbon Hybrid Composite Plates. Polym. Test..

[33] Fiore V., Valenza A., Di Bella G. (2012). Mechanical Behavior of Carbon/Flax Hybrid Composites for Structural Applications. J. Compos. Mater..

[34] Fehri M., Ragueh R.R., Vivet A., Dammak F., Haddar M. (2016). Improvement of Natural Fiber Composite Materials by Carbon Fibers. J. Renew. Mater..

[35] Nisini E., Santulli C., Liverani A. (2016). Mechanical and Impact Characterization of Hybrid Composite Laminates with Carbon, Basalt and Flax Fibres. Compos. Part B Eng..

[36] Issa H., Robin G., Duigou L., Cadou J.M., Guevel Y., Daya E.M. Experimental Investigation of the Effect of Stacking Sequences in Hybrid Carbon/Flax Composites. Proceedings of the International Congress for Applied Mechanics.

[37] Paturel A., Dhakal H.N. (2020). Influence of Water Absorption on the Low Velocity Falling Weight Impact Damage Behaviour of Flax/Glass Reinforced Vinyl Ester Hybrid Composites. Molecules.

[38] Heckadka S.S., Ballambat R.P., Manjeshwar V.K., Kumar M., Hegde P., Kamath A. (2020). Influence of Stack Sequence on the Mechanical Characteristics of Hybrid Composites Analyzed Using Cone Beam Computed Tomography and Scanning Electron Microscopy. Polym. Compos..

[39] Jamshaid H., Ali H., Mishra R.K., Nazari S., Chandan V. (2023). Durability and Accelerated Ageing of Natural Fibers in Concrete as a Sustainable Construction Material. Materials.

[40] Barouni A.K., Dhakal H.N. (2019). Damage Investigation and Assessment Due to Low-Velocity Impact on Flax/Glass Hybrid Composite Plates. Compos. Struct..

[41] Kim C.-U., Song J.-I. (2020). Effect of Hybrid Reinforcement on the Mechanical Properties of Vinyl Ester Green Composites. Fibers Polym..

[42] Attia M.A., El-Baky M.A.A., Abdelhaleem M.M., Hassan M.A. (2020). Hybrid Composite Laminates Reinforced with Flax-Basalt-Glass Woven Fabrics for Lightweight Load Bearing Structures. J. Ind. Text..

[43] Davies P., Sridharan S. (2008). Review of standard procedures for delamination resistance testing. Delamination Behaviour of Composites.

[44] Arumugam V., Militky J., Tunak M. (2016). In-plane shear behavior of 3D spacer knitted fabrics. J. Ind. Text..

[45] Chaterjee S., Adams D., Oplinger D.W. (1993). Test Methods for Composites, a Status Report Volume III: Shear Test Methods.

[46] Fuqua M., Huo S., Thapa A., Gibbon L., Ulven C.A. Challenges in manufacturing oilseed versus linen flax fiber reinforced composites. Proceedings of the International SAMPE Symposium and Exhibition.

[47] Rong M.Z., Zhang M.Q., Liu Y., Yang C.C., Zeng H.M. (2001). The effect of fiber treatment on the mechanical properties of unidirectional sisal-reinforced epoxy composites. Compos. Sci. Technol..

[48] Monette D., Dumond P., Chikhaoui I., Nichols P., Lemaire E.D. (2020). Preliminary Material Evaluation of Flax Fibers for Prosthetic Socket Fabrication. J. Biomech. Eng..

[49] Dhason R., Roy S., Datta S. (2022). The Influence of Composite Bone Plates in Vancouver Femur B1 Fracture Fixation after Post-Operative, and Healed Bone Stages: A Finite Element Study. Proc. Inst. Mech. Eng. Part H J. Eng. Med..

[50] Bihari A., Gee A., Bougherara H., Brzozowski P., Lawendy A.-R., Schemitsch E.H., Zdero R. (2024). Cytotoxicity of Novel Hybrid Composite Materials for Making Bone Fracture Plates. Biomed. Mater..

[51] Daud S.Z.M., Mustapha F., Adzis Z. (2018). Lightning Strike Evaluation on Composite and Biocomposite Vertical-Axis Wind Turbine Blade Using Structural Health Monitoring Approach. J. Intell. Mater. Syst. Struct..

[52] Hinzmann C., Johansen N.F.-J., Hasager C.B., Holst B. (2024). Towards Greener Wind Power: Nanodiamond-Treated Flax Fiber Composites Outperform Standard Glass Fiber Composites in Impact Fatigue Tests. Compos. Part A Appl. Sci. Manuf..

[53] Yang T., Xiong X., Venkataraman M., Novák J. (2019). Investigation on sound absorption properties of aerogel/polymer nonwovens. J. Text. Inst..

[54] Hess K.M., Srubar W.V. (2015). Mechanical Characterization of Gelatin-Flax Natural-Fiber Composites for Construction. J. Renew. Mater..

[55] Bąk A., Mikuła J., Oliinyk I., Łach M. (2024). Basic Research on Layered Geopolymer Composites with Insulating Materials of Natural Origin. Sci. Rep..

[56] Mak K., Fam A., MacDougall C. (2016). Flax fibre composite sandwich panels for structural applications. Building on our growth opportunities. Proceedings of the Annual Conference—Canadian Society for Civil Engineering.

[57] Mohammed L., Ansari M.N.M., Pua G., Jawaid M., Islam M.S. (2015). A Review on Natural Fiber Reinforced Polymer Composite and Its Applications. Int. J. Polym. Sci..

[58] Lincoln J.D., Shapiro A.A., Earthman J.C., Saphores J.-d.M., Ogunseitan O.A. (2008). Design and Evaluation of Bioepoxy-Flax Composites for Printed Circuit Boards. IEEE Trans. Electron. Packag. Manuf..

[59] Amiri A., Burkart V., Yu A., Webster D., Ulven C. (2018). The Potential of Natural Composite Materials in Structural Design. Sustainable Composites for Aerospace Applications.

[60] Faruk O., Bledzki A.K., Fink H.-P., Sain M. (2012). Biocomposites Reinforced with Natural Fibers: 2000–2010. Prog. Polym. Sci..

[61] Papanicolaou G., Chalkias D., Koutsomitopoulou A. (2016). Low Energy Impact and post-impact Behaviour of Epoxy matrix-woven flax fabric composites. UPB Sci. Bull. Ser. D Mech. Eng..

[62] Flax Weaves Its Way into Cars and Aircraft. https://www.plasticsnews.com/article/20130328/NEWS/130329909/flax-weaves-its-way-into-cars-and-aircraft.

[63] Manivannan J.M., Sathishkumar T.P., Subramani S., Dhairiyasamy R. (2024). Investigation on the Fracture and Creep Behavior of the Synthetic and Natural Fiber Laminate Polymer Composite. Matéria (Rio J. ).

[64] Zouhar J., Slaný M., Sedlák J., Joska Z., Pokorný Z., Barényi I., Majerík J., Fiala Z. (2022). Application of Carbon–Flax Hybrid Composite in High Performance Electric Personal Watercraft. Polymers.

[65] An H., Song Y., Liu L., Meng X. (2021). Experimental Study of the Compressive Strengths of Basalt Fiber-Reinforced Concrete after Various High-Temperature Treatments and Cooling in Open Air and Water. Appl. Sci..

[66] Deng Z., Liu X., Liang N., de la Fuente A., Peng H. (2021). Flexural Performance of a New Hybrid Basalt-Polypropylene Fiber-Reinforced Concrete Oriented to Concrete Pipelines. Fibers.

[67] Mohammad Jani N., Shakir Nasif M., Shafiq N., Holt I. (2020). Experimental Investigation on the Effect of Varying Fiber Mix Proportion on the Mechanical and Thermal Performances of Fiber-Reinforced Self-Compacting Concrete under Hydrocarbon Fire Condition. Appl. Sci..

[68] Xiong X., Yang T., Mishra R., Militky J. (2017). Transport properties of aerogel-based nanofibrous nonwoven fabrics. Fibers Polym..

[69] Dvorkin L., Bordiuzhenko O., Tekle B.H., Ribakov Y. (2021). A Method for the Design of Concrete with Combined Steel and Basalt Fiber. Appl. Sci..

[70] Bakis C., Bank L.C., Brown V., Cosenza E., Davalos J.F., Lesko J.J., Triantafillou T.C. (2002). Fiber-reinforced polymer composites for construction-state-of-the-art review. J. Compos. Constr..

[71] (2022). Standard Test Method for Short-Beam Strength of Polymer Matrix Composite Materials and Their Laminates.

[72] Fan Z., Santare M.H., Advani S.G. (2007). Interlaminar Shear Strength of Glass Fiber Reinforced Epoxy Composites Enhanced with Multi-Walled Carbon Nanotubes. Compos. Part A Appl. Sci. Manuf..

[73] Petrucci R., Santulli C., Puglia D., Sarasini F., Torre L., Kenny J.M. (2013). Mechanical Characterisation of Hybrid Composite Laminates Based on Basalt Fibres in Combination with Flax, Hemp and Glass Fibres Manufactured by Vacuum Infusion. Mater. Des..

[74] Barouni A., Lupton C., Jiang C., Saifullah A., Giasin K., Zhang Z., Dhakal H.N. (2021). Investigation into the Fatigue Properties of Flax Fibre Epoxy Composites and Hybrid Composites Based on Flax and Glass Fibres. Compos. Struct..

[75] Poole M., Gower M. (2022). Mechanical Characterisation of 3D Fibre-Reinforced Plastic (FRP) Composites.

[76] Padmanabhan K., Kishore J. (1995). Interlaminar shear of woven fabric Kevlar-epoxy composites in three-point loading. Mater. Sci. Eng. A.

[77] Cui W.C., Wisnom M.R., Jones M. (1992). Failure mechanisms in three and four point short beam bending tests of unidirectional glass/epoxy. J. Strain Anal. Eng. Des..

[78] Madsen B., Aslan M., Lilholt H. (2015). Fractographic Observations of the Microstructural Characteristics of Flax Fibre Composites. Compos. Sci. Technol..

[79] Aslan M., Chinga-Carrasco G., Sørensen B.F., Madsen B. (2011). Strength Variability of Single Flax Fibres. J. Mater. Sci..

[80] Beaugrand J., Guessasma S. (2015). Scenarios of Crack Propagation in Bast Fibers: Combining Experimental and Finite Element Approaches. Compos. Struct..

[81] Richely E., Beaugrand J., Coret M., Binetruy C., Ouagne P., Bourmaud A., Guessasma S. (2023). In Situ Tensile Testing under High-Speed Optical Recording to Determine Hierarchical Damage Kinetics in Polymer Layers of Flax Fibre Elements. Polymers.

